# Use of non-contrast Myocardial T1 times to distinguish between fibrosis and normal myocardial tissue, a possible alternative for patients who cannot receive gadolinium-based contrast agents

**DOI:** 10.1186/1532-429X-18-S1-P317

**Published:** 2016-01-27

**Authors:** Karima Addetia, Amita Singh, Keigo Kawaji, Victor Mor-Avi, Roberto Lang, Amit R Patel

**Affiliations:** Cardiology, University of Chicago, Chicago, IL USA

## Background

Delayed washout of Gadolinium (Gd) contrast in the presence of myocardial scar, fibrosis, or infiltration is the basis for late Gd enhancement (LGE) imaging using cardiovascular magnetic resonance (CMR). It is also known that shortening of native, non-contrast myocardial T1 time is proportional to the degree of myocardial damage. We thus hypothesized that, compared to normal myocardium, native, non-contrast myocardial T1 time would be significantly longer in areas that would have greater Gd deposits on LGE sequences. If confirmed, this approach could potentially be used to detect damaged myocardium without contrast enhancement in patients who cannot receive Gd-based contrast agents.

## Methods

18 patients (63 ± 10 years, 15 men) with cardiomyopathy were prospectively imaged using a 1.5-T scanner (Philips). Modified Look-Locker Imaging (MOLLI) was performed for T1 mapping prior to contrast administration. LGE images were obtained 10 minutes after the administration of Gd-based contrast using a T1-weighted gradient echo pulse sequence with a phase sensitive inversion recovery reconstruction (TR 4.5 ms,TE 2.2 ms, TI 250-300 ms, flip angle 30°, flip angle 5°,voxel size 2 × 2 × 10 mm, SENSE factor 2). An inversion time between 250-300 ms was used to achieve nulling of normal myocardium. Both sets of images were acquired in the short axis plane at the left ventricular basal, mid and apical levels. T1 measurements (Medis) were made in triplicate on pre-contrast MOLLI images in a region of interest confirmed to have LGE and in an area of normal myocardium based on post-contrast images. T-tests were used to compare T1 times in areas with and without LGE (Fig).

## Results

The reason for CMR included viability assessment in 11 patients, cardiac amyloid in 2 patients, non-ischemic cardiomyopathy evaluation in 3 patients, heart transplant follow-up in 1 and cardiac mass evaluation in 1. Mean T1 time in myocardial regions with LGE was 1269 ± 167 msec, in non-LGE segments 1032 ± 118 msec and in the myocardial cavity 1644 ± 113 msec (p < 0.001 LGE positive versus LGE negative segments). A difference in T1 times between LGE and non-LGE segments of <5 % was seen in one case of amyloid with diffuse LGE pattern and in one case of mid-wall stripe suggesting that in cases of diffuse and sparse LGE, T1 time may have difficulty in distinguishing between abnormal and normal myocardium.

## Conclusions

Measured T1 is significantly higher in non-contrast myocardium with confirmed LGE deposits than in normal myocardium. Our findings suggest that it may be feasible to use T1 times to identify regions of damaged myocardium in patients who cannot receive myocardial contrast such as in those with renal failure.

**Figure 1:**
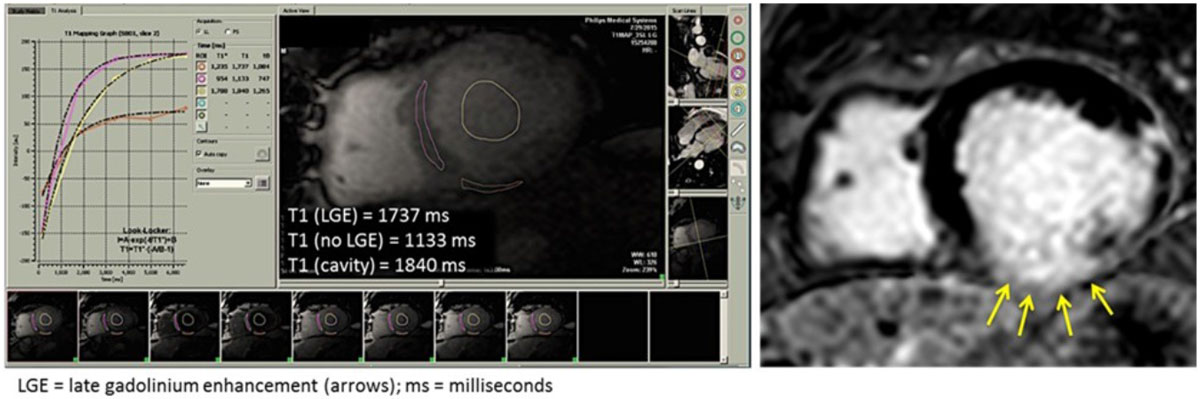
Figure 1

